# Effect of Metabolic Syndrome in the incidence of Rotator Cuff Injury and Recovery following Surgical Repair

**DOI:** 10.26502/jsr.10020438

**Published:** 2025-03-26

**Authors:** Sugeeth Kandikattu, Manas Aavula, Timothy Pisarski, David Parvizi, Devendra K Agrawal

**Affiliations:** Department of Translational Research, College of Osteopathic Medicine of the Pacific, Western University of Health Sciences, Pomona, California 91766 USA

**Keywords:** Apoptosis, Diabetes, Hyperglycemia, Hyperlipidemia, Hypertension, Inflammation, Metabolic syndrome, Obesity, Rotator cuff injury, Tendon re-tear

## Abstract

Rotator cuff injuries are prevalent and have a plethora of risk factors that play a role in both the incidence of injury and recovery from surgical repair. One of the major contributors is metabolic syndrome, which has a variety of different elements that affect the issue under discussion such as hypertension, hyperglycemia, hyperlipidemia, and obesity, which are highly prevalent in patients troubled with this injury. The purpose of this article is to critically review the information from various scientific reports on the underlying cellular and molecular mechanisms of metabolic syndrome increasing the rate of injury and delaying recovery after repair. After critical examination of the findings from many publications in this area, it can be concluded that the factors associated with the metabolic syndrome play a negative role and support the underlying thesis on prolonged recovery with poor outcome following tendon repair in the shoulder. Diabetes Mellitus with elevated insulin levels increases inflammation and cell apoptosis, decreasing healing factors post-surgery. The occurrence of hyperlipidemia and obesity can increase the deposition of xanthomas and the release of adipokines, respectively, which has been proven to delay wound healing as well as increase the risk of reinjury. Studies have also shown that patients with metabolic syndrome who have had a rotator cuff repair tend to have prolonged hospital stays, a higher incidence of reinjury, and increased instances of rehospitalization. The findings together further emphasize the negative effect of metabolic syndrome and how its actions can affect the outcome of the recovery process and lifestyle of a patient post-surgery. These findings further establish the critical consideration of such factors as a cause for incidence, reinjury/rehospitalization, and prolongation of surgical recovery.

## Introduction

Metabolic syndrome is a major health problem with increasing prevalence and incidence all over the world. Indeed, Many factors of metabolic syndrome affect the recovery rate from major reconstructive surgery such as rotator cuff injuries. The most important factors that play a role in the recovery of the musculoskeletal system are obesity, hyperlipidemia, and hyperglycemia, the key factors in the pathogenesis of metabolic syndrome [1].

Obesity is known to increase inflammation in injured areas, which hurts recovery time as well as increases the risk of wound infection [2]. Hyperglycemia can increase the risk of postoperative mortality and cardiovascular, neurological, and infectious morbidity [3]. Hyperlipidemia is the main cause of poor mechanical deficiency, which can lead to impaired muscle-to-tendon healing due to the excessive presence of lipids in the body [4]. The mechanisms of these effects will be further discussed in the background section. Evidence extrapolated from a plethora of articles indicated that these factors play a negative role in the recovery process by increasing the length of postoperative hospital stays, the risk of infection, and the risk of re-injury. Data shows that monitoring these factors is in the best interest of the speed of recovery and decreases complications for these procedures. As these factors continue to increase in prevalence today, it is important to investigate both their individual effects as well as their confounding effects on recovery times from invasive, reconstructive rotator cuff injuries.

## Methods

A research question was formulated to investigate the role of metabolic syndrome in rotator cuff injury. This was followed by the review of literature in Pubmed and Google Scholar between 2005 and 2025, and the published material was selected using several keywords, including diabetes, hyperglycemia, hypertension, metabolic syndrome, obesity, rotator cuff injury, shoulder inflammation, tendon retear, and shoulder stiffness. The title and the abstract of the articles with these keywords were reviewed, and the full article was selected for critical review of the findings based on several criteria, including only English language article, original research article and case reports, relevance to the subject in this article, and proper statistical analyses without any bias. The information from the selected articles was consolidated in a comprehensive manner. Data was then extracted from prevalent literature and used to prove the research question at hand, and it was then analyzed to determine the applicability of the information on the effect of metabolic syndrome in the pathogenesis, incidence, and severity of rotator cuff injury, recovery, and re-tear following tendon repair.

### Metabolic Syndrome and Musculoskeletal System

Metabolic pathways in the body convert food to thermal and chemical energy that tissues can use to do work. Impediments in these pathways can increase the risk for musculoskeletal diseases such as tendinopathy, osteoporosis, osteoarthritis, and more. These diseases can result from systemic inflammation and increased weight bearing due to obesity [5]. Metabolic syndrome encompasses a group of metabolic imbalances that include hypertension, obesity, low HDL cholesterol, hyperglycemia, and hyperlipidemia [5]. Among these, hyperglycemia, obesity, and hyperlipidemia are the major factors affecting the musculoskeletal system. A big reason for the onset of metabolic syndrome is insulin resistance. Insulin is a peptide hormone secreted due to high blood glucose. Insulin resistance causes an increase in free fatty acid (FFA) circulation, which eventually increases cholesterol esters and triglyceride levels and very low-density lipoprotein (VLDL) production [6]. Insulin resistance can stimulate downstream inflammatory pathways such as atherogenesis and tissue fibrosis, causing an increase in inflammatory markers such as IL-6, TNF-α, and C-reactive protein (CRP) [7]. In this critical review article, we will focus on the effects of hyperglycemia and obesity on the incidence of rotator cuff injury and recovery following surgical repair of the tendon.

Wound healing occurs in three main phases: inflammation, proliferation, and remodeling. Hyperglycemia, which is increased blood glucose levels, affects all three phases [8]. It is associated with stiffer blood vessels, causing slower circulation, which leads to decreased oxygenation of tissues. Changes in blood vessels seen in individuals with diabetes are responsible for the decrease in the movement of leukocytes to wound sites, increasing the rate of infections [9]. Wounds in diabetics can also lead to increased inflammation and reduced angiogenesis, which also plays a role in decreased oxygenation. Diabetics have a decrease in crucial proangiogenic factors and an increase in antiangiogenic factors. Non-healing wounds can stay the same for long periods [8]. Endothelial dysfunction, which includes a reduced ability to carry out vasodilation and vascular permeability, is found in hyperglycemic patients. Endothelial cells exposed to hyperglycemia initiate a variety of negative effects, such as an increase in NADPH oxidase (NOX)-derived reactive oxygen species (ROS) [10]. The increase in ROS leads to reduced levels of nitric oxide (NO), causing a decrease in vasodilation, an increase in apoptosis of endothelial cells (EC), and an increase in EC binding to monocytes [11]. Lastly, it has been found that acute hyperglycemia in active young individuals doesn’t affect their muscle strength, power, and endurance, whereas in individuals with chronic hyperglycemia, lower muscular performance was seen, independent of physical activity [12,13].

Obesity is a major health issue that is very apparent within society today. It can be characterized by an increase in adipose stores in the form of brown and white adipose tissue due to a long-term imbalance between calories absorbed and calories expended [14]. Obesity is signified on the BMI scale with a value of ≥ 30 kg/m^2^, severe obesity is ≥ 35 kg/m^2^, and morbid obesity is ≥ 40 kg/m^2^. Obesity can be caused by internal factors, such as genetics, and external factors, such as age, ethnicity, and socioeconomic status. It also plays a major role in the impairment of wound healing. Increased adipose tissue impairs angiogenesis and increases chronic low-grade inflammation. This, in turn, increases the hypoxia-inducible factor 1 alpha (HIF1α) due to impaired oxygenation of adipose tissue, leading to inflammation and fibrosis, decreasing the angiogenic process needed for proper wound healing [15,16]. In addition to this, oxidative stress within obese individuals increases due to a decrease in adiponectin, a cytokine originating from adipose tissue that guards against oxidative stress and inflammation. Adiponectin plays two roles in wound healing. First, it stimulates angiogenesis through an AMP-activated protein kinase pathway [15]. Second, it stimulates the ERK signaling pathway, facilitating keratinocyte growth and migration, which is crucial for the re-epithelialization phase of wound healing. Therefore, the decrease in adiponectin reduces these two processes of wound healing [15]. Oxidative stress also plays a role in tendon degeneration and fibrosis. Hyperlipidemia can inhibit the expression of tendon-related genes within tendon stem cells through the activation of the nuclear factor kappa-B signaling pathway via reactive oxygen species (ROS). This process can trigger apoptosis and autophagy in these cells through AKT/FOXO1 signaling pathway activated by ROS, leading to cellular dysfunction and contributing to tendon degeneration and tendinopathy [17,18].

The rotator cuff is a group of four muscles that are inserted on the superior aspect of the humeral head and originate from the scapula. The four muscles are called supraspinatus, infraspinatus, subscapularis, and teres minor [19]. Rotator cuff tears occur in 20.7% of the population, with 16.9% of people having a tear but not noticing any symptoms. The likelihood of tears increased with age [20]. Patients may notice decreased muscle strength in the abduction and external rotation [21]. A rotator cuff injury encompasses a wide array of injuries ranging from tendinopathy, a tendon that becomes inflamed, to minor tears to full tears. Increasing age is a common cause of rotator cuff injury due to muscle degeneration [22]. Rotator cuff tears are linked with shoulder dysfunction, pain, and weakness. A treatment option can be chosen based on the severity of the symptoms, the cause of the injury, the condition of the muscle-tendon unit, the patient’s ability to go through rehabilitation, and other factors relating to the patient [23].

Hyperlipidemic patients experience increased plasma low-density lipoprotein (LDL) levels, leading to the formation of xanthomas within tendons [24]. LDLs are carrier proteins that include cholesterol, lysolipids, lysophosphatidic acid, modified phospholipids, and isoprostanes [25]. The accumulation of LDL components in the tendon matrix triggers chemokine recruitment and subsequent tissue necrosis. Additionally, hyperlipidemia can promote osteoclast migration at the humeral head, which, along with bone mineralization, weakens tendon insertion strength at the infraspinatus enthesis and impairs the mechanical response of the rotator cuff to loads [26]. Another reason hyperlipidemic patients may experience a higher incidence of rotator cuff tears is due to its effects on collagen. Healthy tendons mainly have type 1 collagen with small amounts of type 3 collagen. Accumulation of cholesterol in tendons in contact with the collagen fibrils can interrupt the synthesis of collagen, harming the structural and biomechanical properties of the tendon [27]. A study looked to analyze the gene and protein expression of rotator cuff tears in hypercholesterolemic patients and healthy patients. Results found that hypercholesterolemic patients had increased levels of inflammatory cytokines and matrix metalloproteinases (MMPs) [27]. There were elevated gene expression levels of IL-6, MMP2, MMP9, and TP53 compared to non-hypercholesterolemic patients, proving there is a distinctive relationship between rotator cuff tears and hypercholesterolemia [28,29]. Similarly, another study found elevated levels of proinflammatory cytokines in diabetic patients, causing tendinopathy. Since diabetic patients are hyperglycemic, tendon cells will respond differently to oxidative stress, causing higher cell death. Advanced glycation end products (AGEs) are found in excessive amounts in patients with diabetes, leading to collagen modifications within tendons and elevated levels of collagen cross-linking. AGEs can elevate bone morphogenetic protein-2 expression in tendon-derived stem cells, potentially hastening the advancement of atherosclerotic calcification [30]. These insights ultimately prove that diabetes contributes to mechanisms underlying impaired bone healing [31].

One study looked at how the biomechanical properties of rotator cuff tendons were affected by hyperlipidemia in swine models. Tests were completed to determine the ultimate tensile strength (UTS) and the modulus of elasticity. Swine that had a hyperlipidemic diet had a lower modulus of elasticity but no difference in UTS in the infraspinatus tendon. The deposition of fat played a major role in the decrease in mechanical properties [32].

Obesity is a major contributor to the likelihood and severity of rotator cuff tears. A study that compared two groups, one with a BMI greater than 25 (group A) and one with a body mass index (BMI) less than 25 (group B), found that individuals with a BMI greater than 30 had the highest chance of a rotator cuff tear. Patients with a large rotator cuff tear had a significantly higher body fat percentage and BMI in comparison to those with a small tear [33].

In a study that looked at the length of recovery in patients who had arthroscopic rotator cuff surgery, diabetic patients had more pain and a much lower range of motion. Diabetic patients saw a plateau in their recovery at 6 months while nondiabetics saw consistent improvement until one year. Diabetes impacts the healing of the tendon-bone junction after rotator cuff arthroplasty. Obese patients had poorer external rotation and function after one year in comparison to nonobese patients [34]. Another study analyzed the metabolic profiles of patients who had a rotator cuff tear accompanied by shoulder stiffness compared to a control group. It was found that the patients had higher levels of sphingolipids within the rotator interval tissue, which is the space surrounded by the subscapularis, supraspinatus, and coracoid serum glycerophospholipid in the anterior capsule tissue of the patient group. Total cholesterol levels in patients with rotator cuff tears and shoulder stiffness were positively correlated to serum glycerophospholipid levels in the anterior capsule [35]. A study demonstrated a notable difference in re-tear between patients with and without hyperlipidemia. Results found that individuals with hyperlipidemia were four times more likely to experience re-tear after rotator cuff repair compared to those without hyperlipidemia. This discovery displays the significant impact of hyperlipidemia on the healing and durability of rotator cuff tissues [36]. A similar study found that the incidence of rotator cuff retear in diabetics increased. The retear rate was notably higher in cases involving diabetes, larger tear sizes, and fatty infiltration [37]. Lastly, an article parallel to these findings conducted a systematic review and found that patients with lipid deposition in rotator cuff tendons had a higher risk of retearing after a rotator cuff repair [38].

Statins, also known as HMG-CoA reductase inhibitors, are the primary medications used to lower lipid levels in medical settings [39]. These drugs block HMG-CoA reductase, an enzyme utilized in cholesterol synthesis. This will reduce cholesterol production and increase the amount of LDL receptors on the cell surface, allowing LDL to be cleared faster from the bloodstream [39]. For example, a study looked to see if there was a causal link between lipid traits and risk for rotator cuff syndrome. It was found that there was no causative link, but there was a significant association between reduced inhibition of HMG-CoA reductase leading to a lesser chance of rotator cuff disease. Increased HMG-CoA expression also led to a lesser chance of rotator cuff disease [40]. One study performed rotator cuff surgery on a rat model of a rotator cuff tear to see the effects of a statin on tendon-bone healing. The statin stimulated the COX2/PGE2/EP4 pathway to promote cell differentiation, migration, and proliferation within tendons. This process enhanced the biochemical strength of the tendon-bone junction [41]. In another study, there was a notable correlation between elevated (moderate & high) perioperative total cholesterol and LDL levels and the likelihood of undergoing revision surgery following an initial arthroscopic rotator cuff repair. The use of statin reduced the necessity for repeat rotator cuff repair [42]. On the other hand, statins have also been shown to have no impact on tendon health. In a study with 77 hyperlipidemic patients who had rotator cuff repairs, 38 patients were using statins while 39 patients were not. Results showed no disparities between the two groups regarding postoperative fatty infiltration, shoulder joint function, and the rates of re-tear [43]. With conflicting evidence like this, more studies need to be done to accurately understand the effects of statin on rotator cuff injuries.

## Key Findings and the Discussion

**Figure 1:**
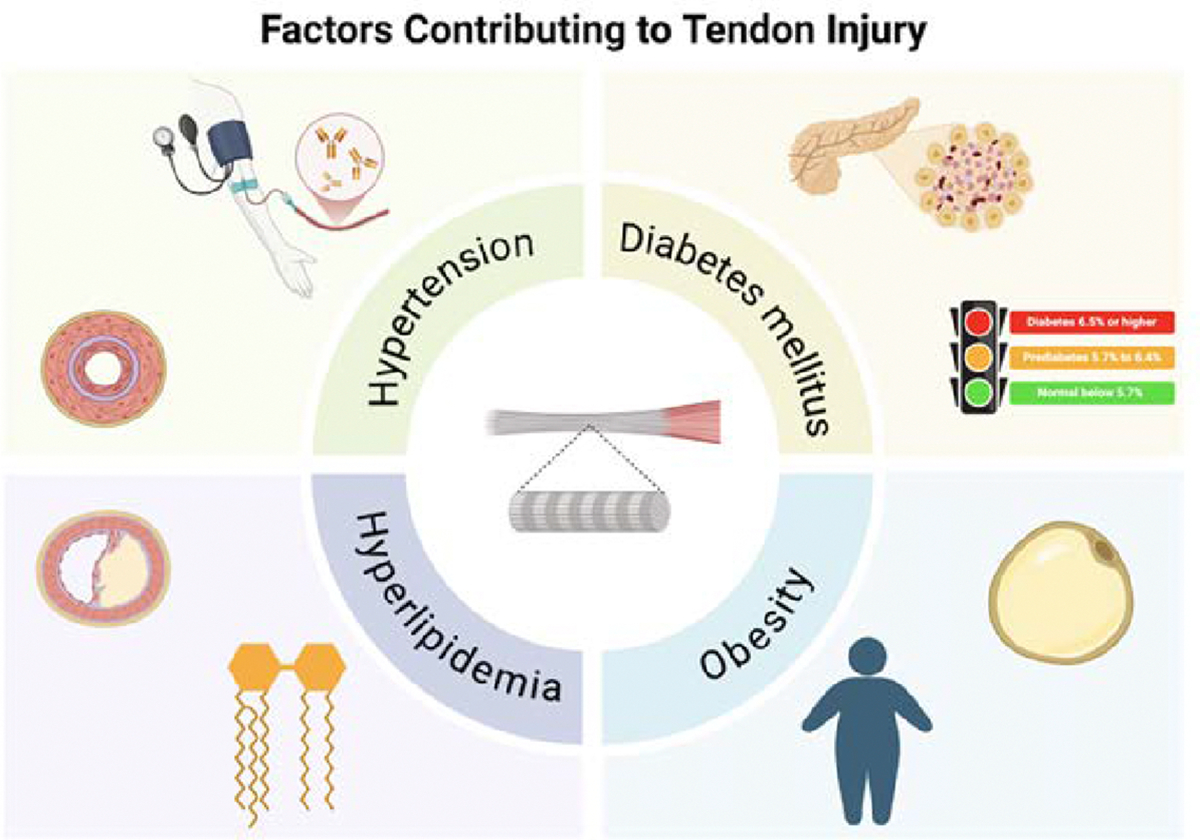
This schematic diagram illustrates that diabetes mellitus, obesity, hyperlipidemia, and hypertension, which are the key factors of metabolic syndrome, play a critical role in increasing the likelihood of tendon injuries through the various mechanisms discussed within the text.

**Figure 2:**
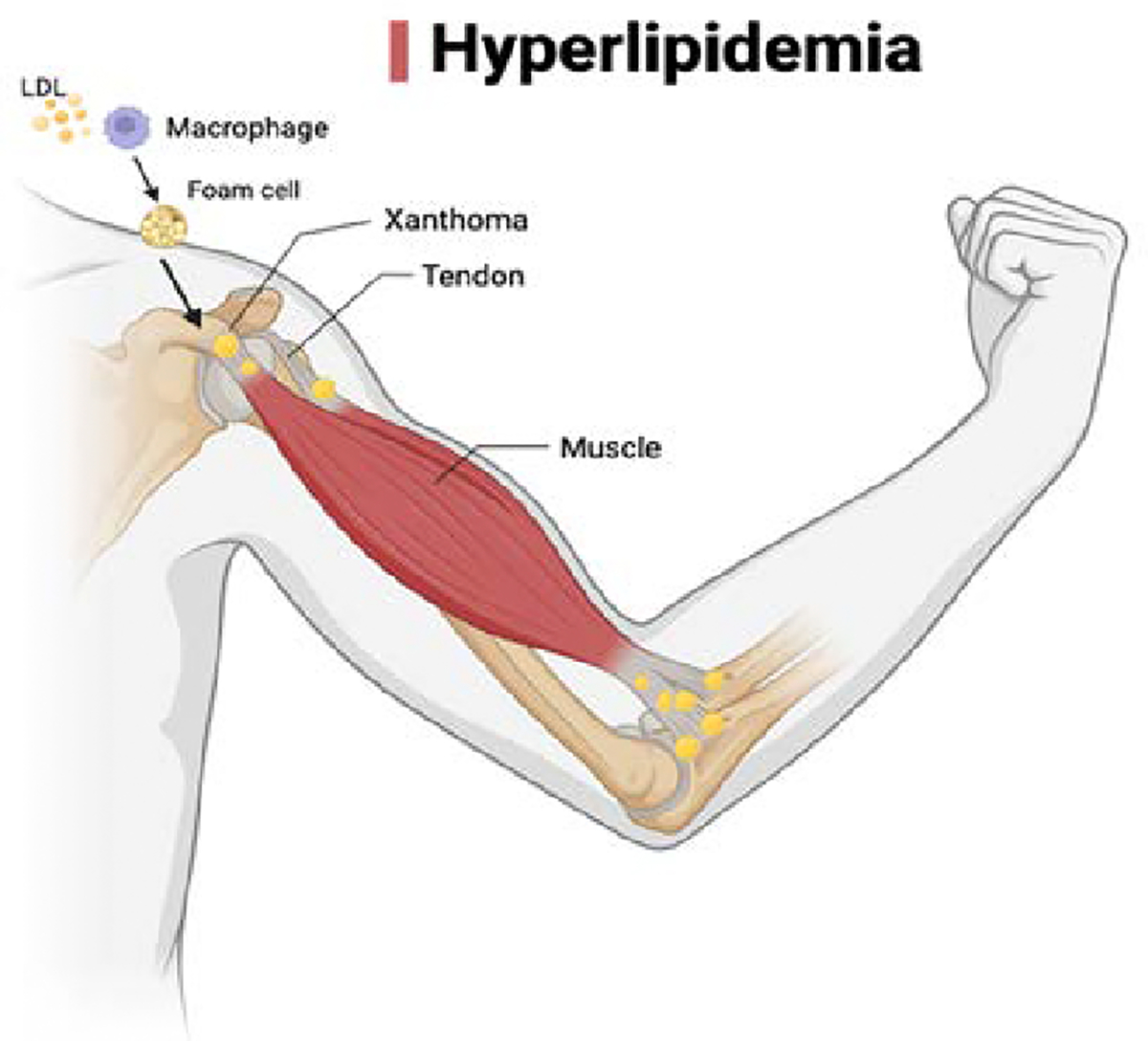
This figure illustrates how hyperlipidemia can affect our tendons and shows the result of continuously elevated cholesterol levels.

As shown in figures 1 and 2, it is important to understand the effects of metabolic syndrome and its various comorbidities on the rotator cuff tendon and the body. It has been proven that diabetes mellitus plays a detrimental role in tendon healing postoperatively, as shown in image one. DM is shown in multiple studies to impair tendon healing by increasing ROS production, increasing HbA1c levels, increasing the risk of infection, and increasing the accumulation of AGEs (Advanced glycation end products) [44]. These various factors restrict the reformation of collagen building blocks used to synthesize/rebuild the tendon, causing poor healing. Obesity is also known to cause pro-inflammatory cytokines and chronic low-grade inflammation, resulting in tendinopathy [45]. It is a major factor in tendon reinjury and hinders recovery [46]. Although hypertension and vascular perfusion of tendons are still being further investigated, high blood pressure generally constricts the size of the blood vessels, which means that there is less perfusion of the tendons [47]. This would mean that due to a lack of blood, oxygen, and nutrient flow, postoperative recovery would be impaired.

**Figure 3:**
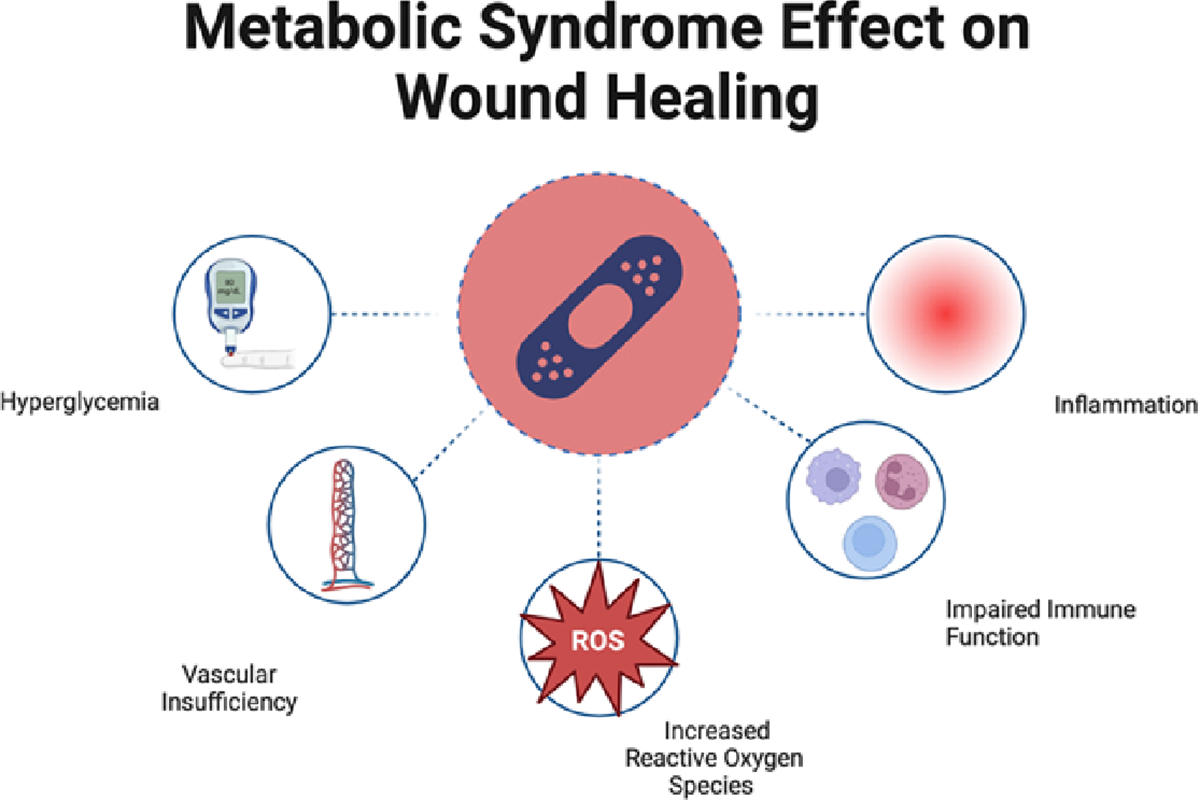
The figure illustrates the various factors that contribute to the prolongation of wound healing. The effects of hyperglycemia, vascular insufficiency, reactive oxygen species (ROS), impaired immune function, and inflammation can be detrimental to the rebuilding and remodeling of injuries such as rotator cuff tears and their repair.

Hyperlipidemia, as illustrated in images one and two, has adverse effects on the tendons both before injury and during the recovery period. In hyperlipidemic patients, there is an increased density of low-density lipoproteins (LDL) and oxidized LDL, which can cause inflammation of the tendon, oxidative stress, and impairment of tendon homeostasis [48,49]. A particular method of action is depicted in figure 3, which shows the native LDL entering the cell and being affected by the cellular oxidants, which are released by the macrophages, releasing oxidized LDL. These oxidized LDLs then accumulate in macrophages, forming foam cells that aggregate into xanthomas forming on the tendon, limiting its function [50].

Figure 3 emphasizes topics discussed in the background. The effects of metabolic syndrome on the prolongation of wound healing led to some of the different consequences, such as prolonged hospital stay, increased chances of rehospitalization, and even death (Figure 3). With increased inflammation, there is likely an increased density of proteases and degenerative substances inhibiting the remodeling occurring during the healing process. Reactive oxygen species play a role in this as well by exacerbating inflammation and increasing oxidative stress. Vascular insufficiency decreases angiogenic processes and limits the diffusion of healing factors to the area, while a deficiency of the immune system can increase the risk of infection. Hyperglycemia plays a strong role in increasing many of the factors above [1,36]. In all, these factors are quite common among individuals with metabolic syndrome, and this is why it is shown that metabolic syndrome has detrimental/prolongation effects on wound healing.

## Conclusion

Even though this topic is still being studied, evidence has been discovered that has been compiled in this paper to support the hypothesis that metabolic syndrome does have a detrimental effect on surgical recovery from RCR and increases the chance of rotator cuff injury. The effects of obesity, hyperlipidemia, hypertension, and diabetes mellitus have been shown to affect the body negatively, and with the information compiled from a plethora of articles, there is convincing information on their negative effects on the rotator cuff tendon both pre/postoperatively. Research has also shown that metabolic syndrome can result in increased postoperative complications and increased risk of injury. It is difficult to apply causation to this thesis since metabolic syndrome occurs with various other comorbidities that can also be detrimental to the rotator cuff tendon as well as the body in general. These challenges are some of the reasons why research is still necessary and are what drive future directions of research on the nuances of RCR and its surgical recovery process.

### Challenges/Gaps in Knowledge

Some challenges faced for this hypothesis were continuously monitoring the high degree of variability of glucose levels, especially with diabetes patients, determining the interrelations between the different factors stated above as well as the effects of various other comorbidities along with their confounding effects and examining how the specific method of repair for the rotator cuff tear could alter the duration of the recovery period and create differing results. When undergoing strenuous surgical procedures, it is common for patients’ blood sugar to increase due to the hypermetabolic stress response, which causes an increase in the release of insulin and can also induce insulin resistance. This is especially true with patients with diabetes mellitus who have poor regulation of insulin, meaning that they will require intensive preoperative, intraoperative, and postoperative care [3]. The challenge arises because tight regulation of glucose levels does not significantly benefit procedure outcomes, and tolerance of moderately elevated glucose levels is common during procedures. In contrast, poor regulation can lead to increased inflammation and delayed recovery, as illustrated in the background. Moderating levels of glucose can have implications to varying degrees on patients, and keeping their level within a certain range can prove to be difficult based on the individual and their specific risk factors.

Aside from diabetes, there are a variety of risk factors that play a role in the incidence and recovery following a rotator cuff injury. Factors such as age, sex, smoking status, and stress can all exacerbate the effects of metabolic syndrome, therefore negatively affecting recovery times and increasing the possibility of injury/re-injury. A major implication of age is that as it increases, the metabolic rate decreases, which can cause complications such as the development of insulin resistance and lead to obesity [51]. Smoking tobacco negatively affects metabolic syndrome by reducing insulin sensitivity and increasing insulin resistance as well as increasing the amount of cortisol and catecholamine, which are known to have insulin-antagonistic properties [52]. Stress can also decrease HDL levels, which in turn can contribute to increased obesity levels and insulin resistance as well [53]. The issue arises with the extent of the effect of the various factors and how the confounding effect of these factors could be skewing data to show prolonged recovery.

Three common methods are used to repair rotator cuff injuries: Traditional open repair, arthroscopic repair, and mini-open repair. For more severe rotator cuff tears, procedures such as superior capsule reconstruction are used [54]. These repair methods have provided similar levels of improvement by the end of the recovery period, but the duration/quality of the recovery period can vary between the different methods. When compared to the other methods of repair, arthroscopic repair shows the lowest possibility of reinjury, the least amount of pain in the region post-surgery, and the most optimal shoulder function [55,56]. Since physicians do not opt for the same method of repair for each type of rotator cuff injury, it is difficult to isolate how the effects of metabolic syndrome could play a role in potentially prolonging the recovery period after varying surgical repair which could affect the recovery duration just as much as the complications of metabolic syndrome [57].

### Future direction

Metabolic syndrome is currently treated with a healthy lifestyle balance, but there has been an investigation into the molecules that affect the hypothalamic signals to the gut as well as targeting the melanocortin system of the brain, which are being looked at for possible targets for drug development [34]. Since the melanocortin system can be used to regulate body weight and promote/exacerbate the comorbidities of obesity, potential targets for this system should be further investigated to confirm treatments that could potentially help improve the recovery process from surgical repair of rotator cuffs and other injuries [58].

As rotator cuff injuries have a high risk of reinjury, new methods of repair are being investigated to decrease the said risk and potentially decrease the occurrences of the comorbidities that occur with prolonged surgery recovery [59,60]. Methods such as stem cell therapies, biological and synthetic patches, and inside arthroscopic treatments are being tested and proven to decrease the time of repair of the injury and the duration of the recovery period [61,62]. Applying these methods to patients with metabolic syndrome should theoretically improve outcomes and decrease the recovery period, but further investigation is needed.

An unfortunate possibility of a rotator cuff injury is a retear, which normally occurs 10% up to 94% of the time in some extreme cases. These reinjuries could be because of several reasons, but due to their prevalence, it substantiates researching whether the repair and recovery could be causing patients to develop metabolic syndrome. This is common with multiple types of injuries, and it is important to investigate because of metabolic syndrome’s effect on recovery from injury, as discussed in this paper [63]. It is also important to understand their correlation since metabolic syndrome is likely to increase the chance of reinjury due to its effects on the body. Using this information could help doctors prepare their patients and educate them on how proper diet, exercise, and care are pivotal pillars in preventing these problems before their occurrence.

## Key Points

- Metabolic Syndrome and Musculoskeletal Diseases: Metabolic syndrome increases the risk for musculoskeletal diseases like tendinopathy, osteoporosis, and osteoarthritis, mainly due to systemic inflammation and atherosclerotic arteries.
- Role of Insulin Resistance: Insulin resistance, a major factor in metabolic syndrome, raises free fatty acids, cholesterol, and LDL production, triggering inflammatory pathways and tissue fibrosis.
- Hyperglycemia Impairs Wound Healing: Elevated blood glucose impairs wound healing by causing endothelial dysfunction, reduced oxygenation, and decreased immune cell movement to wound sites, leading slower recovery.
- Obesity and Wound Healing: Obesity impairs angiogenesis, increases chronic low-grade inflammation, and decreases adiponectin, a cytokine crucial for wound healing, leading to delayed recovery and fibrosis in tendons.
- Hyperlipidemia and Tendon Degeneration: High LDL and oxidative stress in hyperlipidemic individuals can damage tendon cells, promote apoptosis, and disrupt collagen synthesis, weakening tendons and increasing rotator cuff tear risk.
- Rotator Cuff Injuries: The rotator cuff is vulnerable to tears, particularly in older adults. Hyperlipidemia and obesity are linked to an increased incidence and severity of these injuries.
- Impact of Diabetes on Recovery: Diabetic patients face impaired tendon healing after surgery, with higher pain levels, slower recovery, and increased risk of re-tear due to oxidative stress, advanced glycation end products (AGEs), and altered collagen production.
- Obesity and Recovery from Surgery: Obese patients experience poorer outcomes after rotator cuff surgery, including reduced shoulder function, slower recovery, and higher complication rates, likely due to inflammation and metabolic disturbances.
- Challenges and Future Directions: Further research is needed to understand the complex relationship between metabolic syndrome, comorbidities, and rotator cuff injury recovery. New therapies, such as stem cell treatments and biological patches, may benefit patients with metabolic syndrome who are undergoing surgery.
